# Difference in serum complement component C4a levels between hepatitis C virus carriers with persistently normal alanine aminotransferase levels or chronic hepatitis C

**DOI:** 10.3892/mmr.2012.924

**Published:** 2012-05-21

**Authors:** KAZUYUKI IMAKIIRE, HIROFUMI UTO, YUKO SATO, FUMISATO SASAKI, SEIICHI MAWATARI, AKIO IDO, KAZUYA SHIMODA, KATSUHIRO HAYASHI, SHERRI O. STUVER, YOSHITO ITO, TAKESHI OKANOUE, HIROHITO TSUBOUCHI

**Affiliations:** 1 Digestive and Lifestyle Diseases, Department of Human and Environmental Sciences, Kagoshima University Graduate School of Medical and Dental Sciences, Kagoshima; 2 Miyazaki Prefectual Industrial Support Foundation, Miyazaki, Japan; 3 Division of Gastroenterology and Hematology, Internal Medicine, University of Miyazaki, Miyazaki, Japan; 4 Center for Medical Education, Faculty of Medicine, University of Miyazaki, Miyazaki, Japan; 5 Department of Epidemiology, Boston University School of Public Health, Boston, MA, USA; 6 Molecular Gastroenterology and Hepatology, Graduate School of Medical Science, Kyoto Prefectural University of Medicine, Kyoto; 7 Center of Gastroenterology and Hepatology, Saiseikai Suita Hospital, Suita, Japan

**Keywords:** hepatitis C virus, persistent normal alanine aminotransferase, complement component C4a, C4 fragment, serum proteomics

## Abstract

Certain hepatitis C virus (HCV) carriers exhibit persistently normal alanine aminotransferase (ALT) levels (PNALT) (≤30 IU/l) accompanied by normal platelet counts (≥15×10^4^/μl); these individuals show milder disease activity and slower progression to cirrhosis. This study aimed to elucidate the characteristics of HCV carriers with PNALT using serum proteomics. The first group of subjects, who underwent clinical evaluation in the hospital, consisted of 19 HCV carriers with PNALT (PNALT-1) and 20 chronic hepatitis C (CHC-1) patients. The second group of subjects was part of a cohort study on the natural history of liver disease, and included 37 PNALT (PNALT-2) and 30 CHC (CHC-2) patients. Affinity bead-purified serum protein was subjected to matrix-assisted laser desorption ionization time-of-flight mass spectrometry analysis. Serum proteomics showed that 6 protein peaks with mass-to-charge ratios ranging from 1,000 to 3,000 differed significantly between the PNALT-1 and CHC-1 groups. Among these peaks, a 1738-m/z peak protein was identified as a fragment of complement component 4 (C4) and correlated significantly with serum C4a concentrations as determined by enzyme immunoassay. Serum C4a levels were also significantly higher in the PNALT-2 group compared to the CHC-2 group and healthy volunteers. Furthermore, in the PNALT-2 group, serum C4a levels negatively correlated with transaminase levels, but not with other biochemical tests, HCV core antigen levels, peripheral blood cell counts or serum hepatic fibrosis markers. This study indicates that host factors such as C4a not only differ between HCV carriers with PNALT and CHC, but that proteomic approaches could also contribute to the elucidation of factors in PNALT as more differences are discovered.

## Introduction

Hepatitis C virus (HCV) infection, one of the main causes of chronic hepatitis, is estimated to affect 170 million people worldwide (1). The natural history of HCV infection is characterized by acute and eventually chronic infection, and may progress from a long-lasting asymptomatic condition to decompensated liver cirrhosis or hepatocellular carcinoma (HCC) (2). However, the long-term impact of HCV infection is highly variable; some patients with persistent HCV infection exhibit persistently normal alanine aminotransferase (ALT) levels (PNALT), which are associated with milder disease activity and slower progression to cirrhosis (3). In addition, the differences between PNALT patients and those with chronic hepatitis C (CHC) who exhibit elevated ALT levels have not yet been fully elucidated (4).

Persico *et al* reported that the grade of disease activity does not increase over a period of years, and that progression to cirrhosis is slow or absent in patients with HCV-related chronic hepatitis associated with PNALT (5). We have previously reported that the ALT level is a predictor of HCV-associated HCC incidence in a community-based population in Japan (6). In addition, a number of studies have shown that interferon (IFN)-based therapy reduces HCC in patients with CHC, even in those in whom HCV RNA remains detectable (7,8). Continuous normalization of aminotransferase and α-feto protein (AFP) for more than 1 year during IFN therapy is associated with a reduced risk of HCC development following the termination of the IFN therapy (9).

A recent application of proteomic technology has identified a spectral pattern from the serum of patients with liver disease (10–12), and proteomic techniques will be able to identify serum biomarkers that are present in the serum of patients with PNALT. Furthermore, a biomarker or biomarker panel may also help to elucidate a possible mechanism for chronic hepatitis from PNALT and could perhaps lead to the development of more effective treatments for chronic hepatitis. However, proteomic approaches focused on PNALT have not been previously explored.

In this study, we verified differentially expressed protein in serum samples and showed that the level of the complement component 4a (C4a) in serum was higher in HCV carriers with PNALT compared to CHC patients or healthy volunteers. The present study reveals that C4a increases with HCV infection, but decreases with disease progression. Identification of these and other proteins will help clarify the underlying mechanisms and contribute to improved clinical outcomes for HCV carriers.

## Patients and methods

### Study population

Anti-HCV seropositive subjects with detectable HCV core antigen (HCVcAg) or HCV RNA were considered to be persistently infected with HCV and were classified as HCV carriers. ALT levels >30 IU/l and platelet counts <15×10^4^/μl were considered to be abnormal. HCV carriers exhibiting persistently normal ALT levels accompanied by normal platelet counts during the observation period were defined as the PNALT group in this study (13). Subjects who underwent oral or intravenous administration of medical herbs or other palliative therapies were not excluded from this study, but those who had received IFN therapy were excluded. All subjects were negative for hepatitis B virus surface antigen (HBsAg).

The first group of subjects, who were undergoing hospital-based clinical evaluation, consisted of 39 HCV carriers. Of these, 19 with PNALT (PNALT-1 group) and 20 with CHC and abnormal ALT levels (CHC-1 group) were enrolled. HCV carriers with PNALT (PNALT-1 group) were defined as those who had normal serum ALT levels (≤30 U/l) over a 12-month period and on at least 3 different occasions, and platelet counts of ≥15×10^4^/μl. Blood samples from the PNALT-1 and CHC-1 groups were obtained during the last observation period.

The second group of subjects was part of a larger cohort being followed-up as part of a study on the natural history of liver disease; data on these individuals were acquired from 1994 through 2005 (14). An analysis was conducted of HCV carriers who had undergone at least 3 independent ALT measurements obtained during annual general health examinations or liver disease screenings. In total, 37 HCV carriers with persistently normal ALT levels and platelet counts ≥15×10^4^ μl (PNALT-2 group) and 30 HCV carriers with persistently abnormal ALT levels and platelet counts <15×10^4^/μl (CHC-2) were investigated. Blood samples from PNALT-2 or CHC-2 subjects were obtained during the last observation period in this study from 2002 to 2005. Serum samples were also obtained from healthy volunteers without HCV infection (n=12).

After the blood samples were collected, serum was stored at −80°C. Written informed consent was obtained from each subject and the study protocol was approved by the Ethics Committee of Kagoshima University Hospital; the Faculty of Medicine, University of Miyazaki and Kyoto Prefectural Medical School.

### Serum pre-treatment with ClinProt magnetic beads

Serum samples (5 μl) were purified and concentrated using magnetic bead-based weak cation exchange chromatography resins (WCX) (Bluker Daltonics, Bremen, Germany). 2-Cyano-4-hydroxycinnamic acid (CHCA) matrix solution (Bluker Daltonics) was diluted to 0.3 g/l in an ethanol:acetone (2:1) solution. Purified serum samples and diluted CHCA solutions were mixed (1:9), and 1 μl of the solution was applied onto a matrix-assisted laser desorption/ionization time-of-flight (MALDI-TOF) AnchorChip.

### Mass spectrometry (MS) and peptide identification

The AnchorChip target plate was placed in an AutoFlex II TOF/TOF mass spectrometer (Bluker Daltonics). Spectra were acquired in the positive linear mode in a molecular mass range from 1,000 to 3,000 Da. The MALDI-TOF MS spectrum was subjected to a Mascot database search (Matrix Science, Boston, MA, USA) using the SwissProt database.

### Serum markers

The presence of serum anti-HCV antibody (Ab) was determined using a commercially available third-generation enzyme-linked immunosorbent assay (ELISA). Serum levels of HCVcAg were determined by a chemiluminescence enzyme immunoassay (HCV core protein; SRL, Tokyo, Japan), with a detection threshold of 20 fmol/l. The serologically defined HCV genotype (HCV serotype) was tested with a serological genotyping assay kit (Immunocheck F-HCV Grouping, International Reagents Co., Tokyo, Japan). In some patients, the HCV genotype was examined (HCV Core Genotype, SRL, Tokyo, Japan). HCV genotype 1b was included with serotype I, and genotypes 2a and 2b with serotype II. No other HCV genotype was detected in this study population.

The serum concentration of C4a was determined using a C4a ELISA kit (Human C4a ELISA kit, BD Biosciences, San Diego, CA, USA).

### Statistical analysis

The results are presented as the means ± standard deviation (SD). All spectra in MALDI-TOF MS were analyzed using Bluker Daltonics FlexAnalysis 2.2 software and ClinProTools 2.0 software. Statistical analysis of other clinical data was performed using StatView 4.5 software (Abacus Concepts, Berkeley, CA, USA) or SPSS software (SPSS Inc., Chicago, IL, USA). Differences were evaluated by the Mann-Whitney U test, the Fisher’s exact test or the Chi-square test as appropriate. Any probability value <0.05 was considered to indicate a statistically significant difference.

## Results

### Profiling sera from patients with PNALT and chronic hepatitis C using MALDI-TOF MS analysis

In the first hospital-based group, serum levels of ALT, aspartate aminotransferase (AST), and γ-glutamyltranspeptidase (γ-GTP) were lower and platelet counts and total cholesterol were higher in PNALT-1 patients than in CHC-1 patients (Table I). In this group, the sera of patients were analyzed to identify protein peaks that differed most between patient subsets. Serum proteomics revealed that 6 serum protein peaks with mass-to-charge ratios ranging from 1,000 to 3,000 differed significantly between PNALT-1 and CHC-1 groups (Table II). In these protein peaks, a 1738-m/z peak protein was identified as a fragment of C4, with the sequence NGFKSHALQLNNRQI.

### Correlation between the protein peak of 1738 m/z and serum levels of C4a determined by ELISA

**Figure 1:**
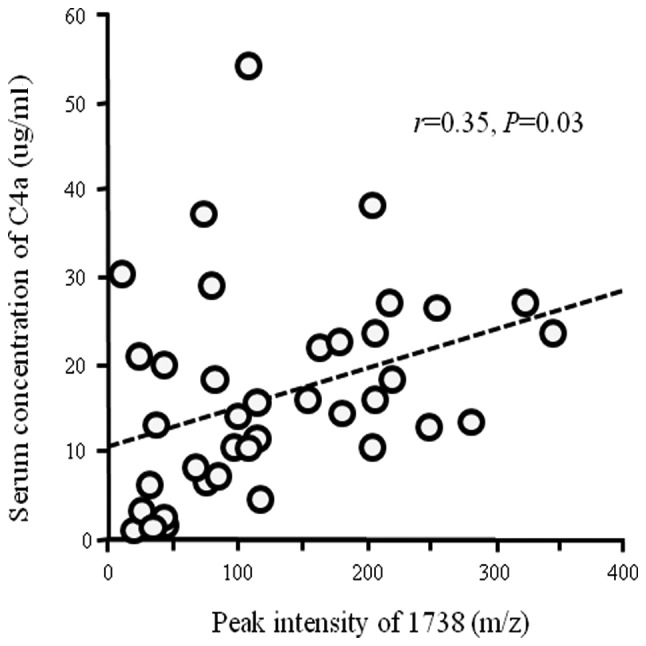
Association between the peak intensity of 1738 m/z (C4 fragment) and the serum level of C4a as determined by enzyme-linked immunosorbent assay. The peak intensity of 1738 m/z correlated with serum C4a levels (r=0.35, P=0.03). C4a, complement component 4a.

Although the identified C4 fragment is part of C4c, serum concentrations of C4c could not be determined by commercially available methods, such as ELISA. By contrast, the recalibrated peak intensity of this fragment significantly correlated with the serum level of C4a, which could be determined with a commercially available assay kit (Fig. 1). In addition, serum concentrations of C4a were significantly higher in PNALT-1 subjects [means ± SD (μg/ml), 20.6±11.9] compared with those in CHC-1 subjects (12.2±10.2) (P=0.01).

### Serum levels of C4a determined by ELISA in the second group

**Figure 2:**
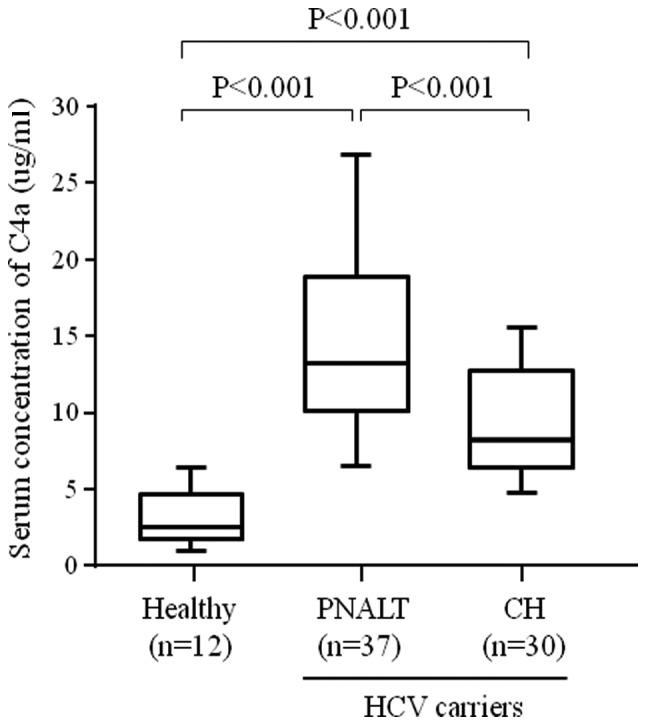
Serum concentrations of C4a determined by enzyme-linked immnosorbent assay in HCV carriers and healthy controls. Serum C4a levels were significantly higher in HCV carriers with PNALT than in HCV carriers with chronic hepatitis or healthy controls (P<0.001). Boxes indicate the median ± 25th percentile, the lower bar indicates the 10th percentile, and the upper bar indicates the 90th percentile. HCV, hepatitis C virus; PNALT, persistently normal alanine aminotransferase; CH, chronic hepatitis; C4a, complement component 4a.

In the cohort-based population, age, the prevalence of females and of serotype II, platelet counts, and serum albumin and total cholesterol levels were significantly higher in the PNALT-2 group than in the CHC-2 group. By contrast, serum AST, ALT and γ-GTP levels were lower in the PNALT-2 group (Table III). In the cohort-based population, serum concentrations of C4a, as determined by ELISA, were significantly higher in the PNALT-2 group than in the CHC-2 group and healthy controls (Fig. 2).

**Figure 3:**
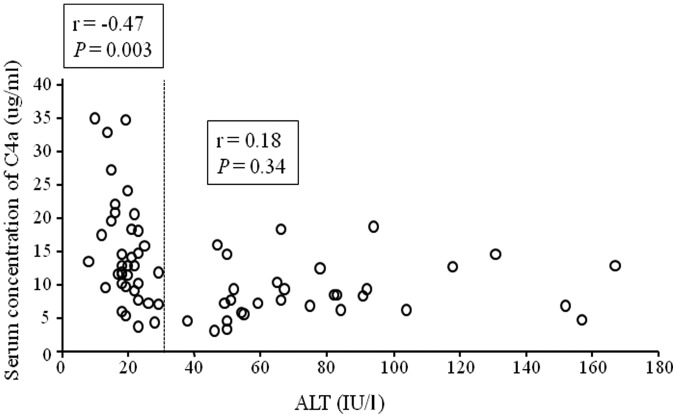
Association between serum C4a and ALT levels in all HCV carriers (including PNALT and chronic hepatitis). Serum C4a levels negatively correlated with serum ALT levels (r=−0.35, P=0.03). This correlation was observed in subjects with normal ALT (≤30 IU/l) (r=−0.47, P=0.003) but not in those with abnormal ALT (>30 IU/l). C4a, complement component 4a; HCV, hepatitis C virus; ALT, alanine aminotransferase; PNALT, persistently normal ALT.

Serum C4a levels in the PNALT-2 group correlated significantly with serum AST and ALT levels, but not with HCVcAg levels or other blood laboratory parameters (Table IV). In addition, a significant negative correlation between serum C4a and ALT levels was observed in the population as a whole (Fig. 3, r=−0.35, P=0.03) and in PNALT patients (Table IV; ALT ≤30 IU/l; Fig. 3), but not in CHC-2 patients (ALT >30 IU/l; Fig. 3).

## Discussion

HCV is not thought to be directly cytopathic to hepatocytes, and a T helper (Th)1-type or cytotoxic T lymphocyte (CTL) response is critically involved in HCV-mediated liver injury (15). Therefore, it is conceivable that various suppressor mechanisms exist against Th1-type immune responses in HCV carriers with PNALT, which may be distinct from those in CHC patients with active liver inflammation (16). However, few studies have focused on PNALT using a serum proteomic approach. In this study of HCV carriers, a number of proteins were detected which were differentially expressed between PNALT and CHC patients. Of these, the C4 fragment was identified by peptide mass fingerprint (PMF) methods following a Mascot search, and serum levels of C4a, which correlated with the protein peak of the identified C4 fragment, were higher in PNALT than in CHC patients, as determined by ELISA. In addition, serum C4a levels correlated with ALT levels in the PNALT but not CHC patients.

Following acute HCV infection, approximately 70% of individuals remain positive for both anti-HCV Ab and HCV RNA, and are defined as HCV carriers. By contrast, approximately 30% of acutely infected individuals clear the HCV and remain positive for anti-HCV Ab but negative for HCV RNA. We confirmed that serum C4a levels in those who cleared the virus were similar to healthy controls (data not shown). Serum C4a levels were higher in CHC patients and individuals with PNALT compared to healthy controls. Therefore, serum C4a levels appear to be at least elevated by existing HCV infection, although serum C4a levels did not correlate with serum HCVcAg levels in individuals with PNALT (Table IV). It was previously reported that serum L-ficolin levels were increased in HCV patients, and that this protein only recognized and bound to glycoproteins E1 and E2 of the HCV envelope, but also activated the complement lectin pathway-mediated cytolytic activity in HCV-infected hepatocytes (17). In the lectin pathway, mannose-binding lectin (MBL)-associated serine protease-2 (MASP-2) cleaves C4, releasing C4a and generating C4b (18). Thus, C4a levels should increase in HCV carriers compared to healthy controls by post-translational mechanisms.

Previous studies have reported decreased serum C4 levels in patients with CHC (19,20). Recently, the HCV core protein and non-structural 5A protein (NS5A) were reported to transcriptionally downregulate C4 expression by modulating the expression of upstream stimulating factor 1 and IFN regulatory factor 1, respectively (21). Thus, serum C4 protein levels are decreased in HCV patients compared to healthy controls as a result of altered transcriptional regulation (22). Although the mechanism of C4a variation in HCV carriers has not been elucidated, our study suggests that serum C4a levels in HCV carriers with PNALT should be dominantly affected by post-translational mechanisms, but patients with CHC may be affected by both translational (downregulation) and post-translational (upregulation) mechanisms.

In CHC, decreased specific C4 activity without C3 consumption suggests complement activation leading to the N-terminal cleavage of C4 with the production of C4a (20). Another study demonstrated increased C4a levels in CHC patients without a significant increase in the levels of C3a (23). Avirutnan *et al* reported that flaviviruses, such as dengue virus, use their non-structural protein, NS1, to attenuate complement activation by directly interacting with C4, leading to viral persistence (24). Although the mechanisms responsible for HCV persistence or PNALT in HCV carriers are not well understood, the interactions between HCV and the host immune system are thought to play a pivotal role in patients with HCV infection.

The majority of individuals with PNALT have minimal or mild inflammation and absent or minimal fibrosis, and follow-up studies have shown disease stability with minimal fibrosis progression over the years, leading to a favorable prognosis. However, cirrhosis and HCC are occasionally observed in HCV carriers with normal ALT levels (25). In addition, some patients with PNALT may develop ALT elevation over time (4), and these individuals may be at increased risk of the significant progression of fibrosis. Long-term observation or liver biopsy has not always been performed; serum C4a levels may be a diagnostic marker for advanced fibrosis or a predictor for ALT elevation in PNALT. These issues should be subject to further analysis.

It has been reported that in HCV carriers with PNALT or normal ALT levels, IFN-based therapy is safe and efficacious (26,27). However, the decision whether or not to treat HCV carriers should be made with the specific clinical setting in mind (28). In addition, it is recommended that serum ALT levels be kept <30 IU/l to prevent the occurrence of HCC (29). If serum C4a levels are an indicator of disease prognosis, HCV carriers with low serum C4a levels may have to be treated despite ALT elevation. More significantly, elucidating the mechanism underlying the association between serum C4a and ALT levels should lead to new approaches to the treatment of HCV carriers with PNALT.

In conclusion, host factors such as C4a differ between HCV carriers with PNALT and CHC patients with elevated ALT levels. Proteomic approaches could greatly contribute to elucidate the host factor in PNALT patients as more differences are discovered. Identification of these and other proteins will help clarify the mechanism and may improve clinical outcomes of HCV carriers.

## Figures and Tables

**Table I:** Patient characteristics in the hospital-based group.

Characteristics	PNALT-1 (n=19)	CHC-1 (n=20)	P-value^a^
Age	55.2±15.1	52.3±11.7	0.17
Gender (male/female)	3/16	7/13	0.27
HCV core antigen (fmol/l)	1163±803	1072±669	0.72
HCV serotype (I/II/UD)	9/5/5	11/5/4	0.87
Platelet count (x10^4^/μl)	21.3±5.5	14.8±4.0	<0.001
AST (IU/l)	25.4±3.9	73.6±37.5	<0.001
ALT (IU/l)	23.5±5.1	90.9±54.8	<0.001
γ-GTP (IU/l)	18.6±8.5	89.4±77.9	<0.001
Total cholesterol (mg/dl)	200.0±24.4	180.1±27.4	0.03
Albumin (g/dl)	4.5±0.2	4.5±0.4	0.76

PNALT, persistently normal ALT; CHC, chronic hepatitis C; n, number of patients; HCV, hepatitis C virus; UD, undetermined; AST, aspartate aminotransferase; ALT, alanine aminotransferase; γ-GTP, γ-glutamyltranspeptidase. Data are presented as the means ± standard deviation or number.

a Differences between mean values were evaluated using either the Fisher’s exact test, the Chi-square test or the Mann-Whitney U test, as appropriate.

**Table II:** Protein peaks expressed differentially between HCV carriers with persistent normal ALT levels (PNALT) and chronic hepatitis C (CHC) patients with abnormal ALT levels.

	Peak intensity		
		
m/z	PNALT-1 (n=19)	CHC-1 (n=20)	P-value^a^
1738	109.4±67.1	83.9±54.0	<0.01
1896	105.0±64.8	111.3±63.3	<0.05
1943	191.3±149.5	139.6±73.6	<0.01
2858	104.0±34.3	85.3±25.6	<0.001
2928	31.8±9.7	64.0±28.9	<0.001
2947	59.3±34.9	80.7±36.2	<0.001

Data are presented as the means ± standard deviation.

a Differences between mean values were evaluated using the Mann-Whitney U test ALT, alanine aminotransferase.

**Table III:** Patient characteristics in cohort-based population with HCV infection.

Characteristics	PNALT-2 (n=37)	CHC-2 (n=30)	P-value^a^
Age	75.6±6.5	70.4±6.6	<0.01
Gender (male/female)	8/29	15/15	0.02
HCV core antigen (fmol/l)	6,042±4,295	4,553±3,546	0.27
HCV serotype (I/II)	14/23	24/6	<0.001
Platelet count (x10^4^/μl)	22.3±5.3	11.8±3.8	<0.001
AST (IU/l)	28.3±8.0	100.1±81.8	<0.001
ALT (IU/l)	19.5±6.0	96.9±81.8	<0.001
γ-GTP (IU/l)	15.1±10.6 (n=31)	56.2±46.7 (n=20)	<0.001
T-Cho (mg/dl)	181.8±29.3 (n=31)	158.8±27.1 (n=29)	0.02
Albumin (g/dl)	4.4±0.3 (n=33)	4.1±0.5 (n=29)	<0.01

HCV, hepatitis C virus; PNALT, persistently normal alanine aminotransferase; CHC, chronic hepatitis C; AST, aspartate aminotransferase; ALT, alanine aminotransferase; γ-GTP, γ-glutamyltranspeptidase; T-Cho, total cholesterol; n, number of patients or the number of samples analyzed. Data are presented as the means ± standard deviation or number.

a Differences between mean values were evaluated using either the Fisher’s exact test or the Mann-Whitney U test, as appropriate.

**Table IV:** Correlation between serum C4a levels and blood laboratory parameters in PNALT subjects.

Parameter	Correlation coefficient	P-value^a^
HCV core antigen	0.06	0.73
White blood cell	−0.06	0.72
Hematocrit	−0.06	0.12
Platelet	0.12	0.51
Albumin	0.03	0.88
γ-globulin	−0.05	0.77
AST	−0.39	0.02
ALT	−0.47	<0.01
Total-bilirubin	−0.07	0.69
Total cholesterol	0.05	0.76
Ferritin	−0.18	0.30
Hyaluronic acid	−0.23	0.17
Type IV collagen	−0.04	0.82
α-fetoprotein	−0.29	0.09
DCP	−0.07	0.69

PNALT, persistently normal alanine aminotransferase; HCV, hepatitis C virus; AST, aspartate aminotransferase; ALT, alanine aminotransferase; DCP, des-γ-carboxy prothrombin.

a P-values were assessed by Spearman’s rank correlation analysis.
